# Secondary risks of bleeding and associated outcome after tirofiban application during interventional treatment of intracranial aneurysms

**DOI:** 10.1016/j.bas.2026.106125

**Published:** 2026-06-11

**Authors:** Michael Griessmair, Jannis Bodden, Dennis M. Hedderich, Jan Kirschke, Christian Maegerlein, Martin Renz, Paul Platzek, Bernhard Meyer, Claus Zimmer, Maria Wostrack, Tobias Boeckh-Behrens, Julian Schwarting

**Affiliations:** a Institute of Diagnostic and Interventional Neuroradiology, TUM University Hospital, School of Medicine and Health, Technical University of Munich, Munich, Germany; b Department of Diagnostic, Interventional and Pediatric Radiology, Inselspital Bern, University of Bern, Switzerland; c Department of Neurosurgery, TUM University Hospital, School of Medicine and Health, Technical University of Munich, Munich, Germany; d Institute for Stroke and Dementia Research (ISD), University Hospital, LMU Munich, Munich, Germany

**Keywords:** Long-term administration of tirofiban, Bleeding safety, Ruptured intracranial aneurysms, Endovascular treatment

## Abstract

**Introduction:**

Device-assisted endovascular treatment of ruptured aneurysms may require antiplatelet therapy, but bleeding risk is a concern. This retrospective propensity score-matched cohort study investigated whether tirofiban, including prolonged administration for ≥7 days, was associated with secondary bleeding complications.

**Methods:**

We analyzed 99 patients treated endovascularly aSAB between 2006 and 2024, including 51 patients receiving tirofiban and 48 controls selected using propensity score matching. Tirofiban patients were categorized into short-term treatment (<7 days) and prolonged treatment (≥7 days). Bleeding complications were classified using an adapted Heidelberg bleeding classification and categorized as spontaneous or surgery-associated.

**Results:**

For the main bleeding endpoints, evaluable data were available for 42 controls, 29 patients with short-term tirofiban, and 18 patients with prolonged tirofiban. Any spontaneous secondary bleeding occurred in 6/42 controls (14%), 2/29 short-term tirofiban patients (7%; p = 0.46), and 1/18 prolonged tirofiban patients (6%; p = 0.66). Surgery-associated bleeding occurred in 13/42 controls (31%), 11/29 short-term tirofiban patients (38%; p = 0.72), and 7/18 prolonged tirofiban patients (39%; p = 0.77). Clinically relevant bleeding occurred in 2/42 controls (5%), 0/29 short-term tirofiban patients (0%; p = 0.51), and 1/18 prolonged tirofiban patients (6%; p = 1.00). Median hospital duration was 21 days in controls, 18 days in short-term, and 31 days in prolonged tirofiban patients.

**Conclusion:**

In this small retrospective cohort, tirofiban, including prolonged administration for ≥7 days, was not associated with a statistically significant increase in spontaneous, surgery-associated, or clinically relevant secondary bleeding. These exploratory findings require confirmation in larger prospective studies.

## Highlights

• Tirofiban after endovascular treatment of ruptured intracranial aneurysms showed no significant increase in secondary bleeding.
• Prolonged administration ≥7 days did not increase spontaneous, surgical, or clinically relevant bleeding.
• Hospital stay was numerically longer in the prolonged group, possibly due to higher clinical/procedural complexity.

## 1 Introduction

Aneurysmal subarachnoid hemorrhage (aSAH) is a severe form of hemorrhagic stroke and remains associated with substantial morbidity and mortality despite advances in aneurysm treatment and neurocritical care (Schatlo et al., 2021). Definitive aneurysm occlusion is required to prevent rebleeding. The choice between endovascular and microsurgical treatment depends on multiple clinical and anatomical factors, including neurological status, hemorrhage burden, aneurysm morphology and location, comorbidities, and local treatment expertise (Hoh et al., 2023).

Endovascular therapy has become a central treatment strategy for ruptured intracranial aneurysms. While many ruptured aneurysms can be treated by simple coiling, complex aneurysm configurations may require adjunctive devices such as stents, flow diverters, or intrasaccular flow-disruption devices (Limaye et al., 2019). These techniques may expand endovascular treatment options in selected complex cases but are associated with an increased risk of thromboembolic complications. Therefore, peri- and postinterventional antiplatelet therapy is often required when intravascular devices are used (Zanaty et al., 2021).

Antiplatelet therapy in the acute phase after aSAH is challenging. Patients with ruptured aneurysms frequently require additional invasive procedures during the early neurocritical care period, including external ventricular drainage, ventriculoperitoneal shunt placement, decompressive craniectomy, or other neurosurgical interventions. In this setting, platelet inhibition may increase concern for secondary hemorrhagic complications (Zanaty et al., 2020), (Chalouhi et al., 2012), (Kim et al., 2016). These concerns can influence interdisciplinary treatment decisions, particularly in complex ruptured aneurysms in which device-assisted endovascular treatment is considered. However, treatment selection remains multifactorial, and endovascular treatment is increasingly used whenever anatomically and clinically appropriate.

Tirofiban is a reversible glycoprotein IIb/IIIa receptor antagonist with rapid onset and a short plasma half-life. These pharmacological properties make it attractive in neurointerventional procedures requiring fast and controllable platelet inhibition, especially when subsequent invasive procedures may become necessary. Compared with oral antiplatelet agents, tirofiban may allow more flexible peri-procedural management because platelet inhibition can be discontinued more rapidly. Other intravenous antiplatelet agents, including eptifibatide, abciximab, and the P2Y12 inhibitor cangrelor, are also used in selected neurointerventional settings; however, the present study focuses specifically on tirofiban.

Previous studies have primarily assessed short-term peri-interventional tirofiban administration in selected patients with ruptured intracranial aneurysms (Hudson et al., 2018), (Hudson et al., 2019), (Cagnazzo et al., 2020), (Scholz et al., 2013). In contrast, evidence on prolonged intravenous tirofiban administration during the early neurocritical care phase remains limited, particularly in patients requiring device-assisted aneurysm treatment and additional invasive procedures. This issue is clinically relevant because transition to oral dual antiplatelet therapy may be delayed in complex cases due to anticipated secondary procedures, uncertain enteral absorption, fluctuating clinical status, or the need for reversible platelet inhibition.

In our institutional practice, a treatment duration of 7 days was used as a pragmatic threshold to distinguish short-term from prolonged tirofiban administration. This cutoff was chosen to reflect the early high-risk phase after aSAH, during which secondary neurosurgical and neurocritical care interventions are frequently required. Nevertheless, this threshold is clinically defined and should not be interpreted as a biologically validated cutoff.

Therefore, the aim of this retrospective propensity score-matched single-center cohort study was to investigate whether tirofiban administration after endovascular treatment of ruptured intracranial aneurysms between 2006 and 2024 was associated with secondary intracranial bleeding complications. In particular, we assessed whether short-term tirofiban administration (<7 days) or prolonged tirofiban administration (≥7 days) was associated with spontaneous, surgery-associated, or clinically relevant bleeding or longer hospitalization compared with matched controls without tirofiban. Given the long study period, potential changes in endovascular devices, peri-procedural antiplatelet management, imaging protocols, and neurocritical care were considered relevant when interpreting the findings.

## 2 Methods

### 2.1 Study design and patient selection

This retrospective single-center cohort study was conducted at the Department of Diagnostic and Interventional Neuroradiology, Technical University of Munich. We reviewed patients who underwent endovascular treatment for aneurysmal subarachnoid hemorrhage due to a ruptured intracranial aneurysm between 2006 and 2024. The study was designed and reported in accordance with the STROBE guidelines for observational studies (von Elm et al., 2007).

Patients were eligible for the tirofiban cohort if aneurysm rupture was verified by non-contrast CT, CTA, or DSA and if peri- and/or postinterventional tirofiban was administered because of device-assisted aneurysm treatment, periprocedural thromboembolic complications, or increased thromboembolic risk. Patients treated endovascularly during the same period without tirofiban served as potential controls and were selected by propensity score matching.

The final cohort included 99 patients, comprising 51 patients treated with tirofiban and 48 controls without tirofiban. Within the tirofiban cohort, 33 patients received short-term tirofiban administration and 18 patients received prolonged tirofiban administration. For the main bleeding endpoint analysis, evaluable data were available for 42 patients in the no-tirofiban group, 29 patients in the short-term tirofiban group, and 18 patients in the prolonged tirofiban group. Denominators differed slightly between individual outcomes according to the availability of evaluable observations. Entries coded as missing or not evaluable were excluded from the denominator of the respective endpoint.

### 2.2 Tirofiban administration

The indication for aneurysm occlusion strategy and tirofiban administration was determined by interdisciplinary consensus between neurointerventionalists and neurosurgeons. Tirofiban was administered in patients undergoing device-assisted endovascular treatment, including stent-assisted coiling, flow-diverter implantation, or intrasaccular devices, and in cases with periprocedural thromboembolic complications or increased thromboembolic risk.

Short-term tirofiban administration was defined as treatment for <7 days, whereas prolonged tirofiban administration was defined as treatment for ≥7 days. This threshold was chosen as a pragmatic clinical cutoff reflecting the early high-risk phase after aneurysmal subarachnoid hemorrhage, during which secondary neurosurgical and neurocritical care procedures are frequently required. The cutoff was not intended to represent a biologically validated threshold.

Detailed data on transition from intravenous tirofiban to oral antiplatelet therapy, including exact timing of conversion, oral loading regimens, and platelet function testing, were not consistently available across the retrospective study period and therefore could not be systematically analyzed.

### 2.3 Clinical and radiological data

Clinical and radiological data were extracted from electronic medical records, procedural reports, intensive care documentation, neurosurgical reports, radiology reports, and the institutional imaging archive. Baseline clinical severity was assessed using the Hunt and Hess scale, and the extent of subarachnoid hemorrhage was graded using the modified Fisher scale. Aneurysm location was categorized as anterior or posterior circulation.

Additional neurosurgical procedures after aneurysm treatment were recorded, including external ventricular drainage, ventriculoperitoneal shunt placement, and decompressive craniectomy. Duration of hospitalization was recorded in days. Early mortality was defined as death within 30 days after aneurysm rupture.

### 2.4 Outcome measures and bleeding classification

The primary outcome was the occurrence of secondary intracranial bleeding complications after endovascular treatment. Bleeding events were categorized as spontaneous secondary bleeding or surgery-associated secondary bleeding. Surgery-associated bleeding was defined as hemorrhage occurring along the surgical tract or in temporal association with an invasive neurosurgical procedure, including external ventricular drainage, ventriculoperitoneal shunt placement, or decompressive craniectomy.

**Fig. 1:**
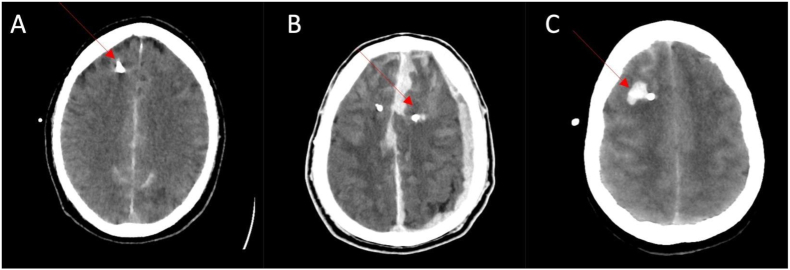
Characterization of bleeding in accordance with the Heidelberg Bleeding Classification A: HI1 scattered small petechiae without mass effect B HI2 Confluent petechiae without mass effect C: PH1: Intraparenchymal bleeding without mass effect.

Bleeding complications were classified using an adapted version of the Heidelberg bleeding classification (von Kummer et al., 2015). The classification was modified for the present aneurysmal subarachnoid hemorrhage cohort to capture petechial hemorrhage, parenchymal hemorrhage, subarachnoid rebleeding, and subdural hematoma. HI1 was defined as scattered small petechial hemorrhage without mass effect. HI2 was defined as confluent petechial hemorrhage without mass effect. PH1 was defined as localized parenchymal hemorrhage without clinically relevant mass effect. PH2 was defined as parenchymal hemorrhage with space-occupying effect or clinically relevant mass effect (see Fig. 1). SAH was defined as secondary subarachnoid hemorrhage or aneurysm rebleeding. SDH was defined as subdural hematoma.

Clinically relevant bleeding was defined as any secondary intracranial bleeding event requiring specific treatment, as documented in the clinical records.

Secondary outcomes were duration of hospitalization and 30-day mortality. Procedural success was not included as an outcome because no uniform definition was available across the complete retrospective study period.

### 2.5 Propensity score matching and covariate balance

Propensity score matching was performed to reduce baseline differences between patients treated with tirofiban and patients not treated with tirofiban. The propensity score model included baseline variables that were available across the complete study period and were considered clinically relevant for hemorrhage severity and bleeding risk: sex, Hunt and Hess grade, modified Fisher grade, aneurysm location categorized as anterior or posterior circulation, pre-existing oral anticoagulation, and pre-existing antiplatelet therapy.

After matching, covariate balance between groups was assessed using standardized mean differences rather than relying solely on significance testing. For binary and continuous variables, standardized mean differences were calculated using conventional definitions. For multi-level categorical variables, the maximum absolute category-specific standardized mean difference was reported. P-values for baseline comparisons were provided descriptively but were not used as the primary measure of covariate balance.

Residual imbalance after matching was considered when interpreting the results. Because of the retrospective design, small sample size, and limited availability of structured data across the long study period, not all potentially relevant confounders could be incorporated into the propensity score model. In particular, aneurysm size and morphology, exact device type, treatment complexity, treatment era, vasospasm or delayed cerebral ischemia, timing and number of secondary neurosurgical procedures, and details of antiplatelet transition strategies were not uniformly available. These variables were therefore considered potential sources of residual confounding.

### 2.6 Statistical analysis

Statistical analyses were performed using Python 3.8 with pandas and scipy.stats. Continuous variables were summarized as median with interquartile range or mean ± standard deviation, depending on distribution and reporting context. Categorical variables were summarized as absolute numbers and percentages.

For categorical outcomes, Fisher's exact test was used when expected cell counts were small; otherwise, chi-square testing was applied. Continuous variables were compared using the Mann–Whitney *U* test or independent-samples *t*-test, as appropriate. Hospital duration was reported as median [interquartile range] and compared using the Mann–Whitney *U* test.

Bleeding complications were compared between controls, patients with short-term tirofiban administration, and patients with prolonged tirofiban administration. Pairwise comparisons were performed for short-term tirofiban versus no tirofiban and prolonged tirofiban versus no tirofiban. Exact p-values were reported where applicable. P-values were not calculated when no events occurred in either comparison group.

Because of the small sample size and low number of events, analyses of bleeding outcomes were considered exploratory. Non-significant results were not interpreted as evidence of equivalence or proof of safety. A p-value <0.05 was considered statistically significant. No formal power calculation or equivalence analysis was performed.

### 2.7 Ethical approval

The study was approved by the local Research Ethics Committee of the Technical University of Munich (186/20S). Because this was a retrospective analysis of routinely collected data and the included data did not allow identification of individual patients, the requirement for retrospective informed consent was waived by the ethics committee. All methods were performed in accordance with relevant guidelines and regulations.

## 3 Results

### 3.1 Baseline characteristics

A total of 99 patients with aneurysmal subarachnoid hemorrhage due to a ruptured intracranial aneurysm were included in the final cohort. Of these, 51 patients received peri- and/or postinterventional tirofiban, and 48 patients did not receive tirofiban.

Baseline characteristics and covariate balance are summarized in Table 1. Median age was 57.2 years [IQR, 48.3–69.0] in the tirofiban group and 55.7 years [IQR, 48.3–66.7] in the no-tirofiban group. Female patients accounted for 34/51 patients (67%) in the tirofiban group and 30/48 patients (62%) in the no-tirofiban group. Aneurysms were predominantly located in the anterior circulation in both cohorts. Standardized mean differences showed residual imbalance for some baseline variables, including Hunt and Hess grade, modified Fisher grade, aneurysm site, and ventriculoperitoneal shunt placement.

**Table 1:** **Baseline characteristics and covariate balance of patients with and without tirofiban.** Values are presented as median [IQR] for age and n (%) for categorical variables. SMD = standardized mean difference. For multi-level categorical variables, the maximum absolute category-specific SMD is shown. Fisher's exact test was used for 2x2 comparisons with small expected cell counts; otherwise, chi-square tests were used.

Parameter	Category	Tirofiban n = 51	No tirofiban n = 48	Total n = 99	p-value	SMD
Age		57.2 [48.3, 69.0]	55.7 [48.3, 66.7]	55.9 [48.2, 67.6]	0.508	0.229
Female sex		34 (67%)	30 (62%)	64 (65%)	0.823	0.087
Hunt and Hess	1	6 (12%)	6 (12%)	12	0.142	0.374
	2	23 (45%)	15 (31%)	38		
	3	2 (4%)	7 (15%)	9		
	4	12 (24%)	7 (15%)	19		
	5	8 (16%)	13 (27%)	21		
Modified Fisher	1	3 (6%)	2 (4%)	5	0.477	0.311
	2	2 (4%)	2 (4%)	4		
	3	23 (45%)	29 (60%)	52		
	4	23 (45%)	15 (31%)	38		
Aneurysm site	Anterior circulation	34 (67%)	37 (77%)	71 (72%)	0.354	0.233
	Posterior circulation	17 (33%)	11 (23%)	28		
Surgeries after intervention	External ventricular drainage	37 (73%)	36 (75%)	73 (74%)	0.961	0.056
	Ventriculoperitoneal shunt	7 (14%)	12 (25%)	19 (19%)	0.243	0.288
	Decompressive craniectomy	4 (8%)	2 (4%)	6 (6%)	0.679	0.155

Additional neurosurgical procedures after aneurysm treatment were common in both groups. External ventricular drainage was performed in 37/51 patients (73%) in the tirofiban group and 36/48 patients (75%) in the no-tirofiban group. Ventriculoperitoneal shunt placement was performed in 7/51 patients (14%) in the tirofiban group and 12/48 patients (25%) in the no-tirofiban group. Decompressive craniectomy was performed in 4/51 patients (8%) in the tirofiban group and 2/48 patients (4%) in the no-tirofiban group (see Table 1).

### 3.2 Bleeding endpoint population

The bleeding endpoint analysis included patients with evaluable bleeding outcome data. Entries coded as missing or not evaluable were excluded from the respective endpoint denominator. For the main bleeding endpoints, evaluable data were available for 42 patients in the no-tirofiban group, 29 patients in the short-term tirofiban group, and 18 patients in the prolonged tirofiban group.

### 3.3 Secondary bleeding complications

Spontaneous secondary bleeding events were infrequent and did not differ significantly between groups. Any spontaneous secondary bleeding occurred in 6/42 patients (14%) in the no-tirofiban group, 2/29 patients (7%) in the short-term tirofiban group (p = 0.46 vs. control), and 1/18 patients (6%) in the prolonged tirofiban group (p = 0.66 vs. control). No spontaneous PH2 hemorrhage occurred in any group.

Surgery-associated secondary bleeding occurred in 13/42 patients (31%) in the no-tirofiban group, 11/29 patients (38%) in the short-term tirofiban group (p = 0.72 vs. control), and 7/18 patients (39%) in the prolonged tirofiban group (p = 0.77 vs. control). Individual surgery-associated bleeding subtypes, including HI1, HI2, PH1, PH2, SAH/rebleeding, and SDH, did not differ significantly between tirofiban subgroups and controls.

Clinically relevant bleeding occurred in 2/42 patients (5%) in the no-tirofiban group, 0/29 patients (0%) in the short-term tirofiban group (p = 0.51 vs. control), and 1/18 patients (6%) in the prolonged tirofiban group (p = 1.00 vs. control) (see Table 2).

**Table 2:** **– Secondary bleeding complications and clinical outcomes.** Values are n/N (%) unless otherwise indicated. Denominators reflect available evaluable observations. P-values compare each tirofiban subgroup with controls. Fisher's exact or chi-square tests were used for categorical outcomes, as appropriate. Hospital duration is reported as median [IQR] and was compared using the Mann–Whitney *U* test. P-values were not calculated when no events occurred in either group.

Outcome	No tirofiban n/N (%)	Tirofiban <7d n/N (%)	p < 7d vs control	Tirofiban ≥7d n/N (%)	p ≥ 7d vs control
**Spontaneous secondary bleeding**					
HI1	3/42 (7%)	1/29 (3%)	0.64	0/18 (0%)	0.55
HI2	1/42 (2%)	0/29 (0%)	1.00	0/18 (0%)	1.00
PH1	1/42 (2%)	1/29 (3%)	1.00	1/18 (6%)	0.51
PH2	0/42 (0%)	0/29 (0%)	n/a	0/18 (0%)	n/a
SAH/rebleeding	1/42 (2%)	0/29 (0%)	1.00	0/18 (0%)	1.00
SDH	1/42 (2%)	0/29 (0%)	1.00	0/18 (0%)	1.00
**Any spontaneous bleeding**	6/42 (14%)	2/29 (7%)	0.46	1/18 (6%)	0.66
**Surgery-associated secondary bleeding**					
HI1	8/42 (19%)	5/29 (17%)	1.00	3/18 (17%)	1.00
HI2	4/42 (10%)	5/29 (17%)	0.47	2/18 (11%)	1.00
PH1	1/42 (2%)	1/29 (3%)	1.00	2/18 (11%)	0.21
PH2	0/42 (0%)	0/29 (0%)	n/a	0/18 (0%)	n/a
SAH/rebleeding	1/42 (2%)	0/29 (0%)	1.00	0/18 (0%)	1.00
SDH	1/42 (2%)	1/29 (3%)	1.00	2/18 (11%)	0.21
**Any surgery-associated bleeding**	13/42 (31%)	11/29 (38%)	0.72	7/18 (39%)	0.77
Clinically relevant bleeding	2/42 (5%)	0/29 (0%)	0.51	1/18 (6%)	1.00
Hospital duration, days	21 [17, 34]	18 [15, 25]	0.25	31 [23, 41]	0.06
Death within 30 days	2/48 (4%)	4/33 (12%)	0.22	1/18 (6%)	1.00

### 3.4 Hospitalization and early mortality

Hospital duration was numerically longer in the prolonged tirofiban group than in the short-term tirofiban and no-tirofiban groups. Median hospital duration was 31 days [IQR, 23–41] in the prolonged tirofiban group, compared with 18 days [IQR, 15–25] in the short-term tirofiban group and 21 days [IQR, 17–34] in the no-tirofiban group. This difference did not reach statistical significance for prolonged tirofiban versus no tirofiban (p = 0.06) or for short-term tirofiban versus no tirofiban (p = 0.25).

Death within 30 days occurred in 2/48 patients (4%) in the no-tirofiban group, 4/33 patients (12%) in the short-term tirofiban group (p = 0.22 vs. control), and 1/18 patients (6%) in the prolonged tirofiban group (p = 1.00 vs. control) (see Table 2).

## 4 Discussion

Intravascular occlusion methods such as stent-assisted coiling and flow-diverter implantation are increasingly used in selected patients with ruptured intracranial aneurysms, but frequently require platelet aggregation inhibition to prevent thromboembolic complications (Hoh et al., 2023), (Samaniego et al., 2019), (Simonato et al., 2022), (Hasanpour et al., 2024). This is particularly challenging in patients with aneurysmal subarachnoid hemorrhage, who often require additional invasive neurosurgical procedures such as external ventricular drainage, ventriculoperitoneal shunt placement, or decompressive craniectomy during the early neurocritical care phase (Hudson et al., 2018), (Hudson et al., 2019), (Cagnazzo et al., 2020). Therefore, the use of antiplatelet therapy in this setting represents a relevant therapeutic dilemma, balancing thromboembolic protection against the potential risk of secondary hemorrhagic complications.

In this monocentric retrospective propensity score-matched cohort study, we compared patients who received tirofiban after endovascular treatment of a ruptured intracranial aneurysm with matched controls who did not receive tirofiban. The main finding was that tirofiban administration, including prolonged treatment, was not associated with a statistically significant increase in spontaneous, surgery-associated, or clinically relevant secondary intracranial bleeding. In particular, we observed no clear increase in aneurysm re-rupture or spontaneous intraparenchymal hemorrhage. Most bleeding events occurred in association with neurosurgical interventions such as EVD placement, shunt surgery, or craniectomy. Importantly, clinically relevant bleeding was not increased in the tirofiban groups.

These findings are broadly consistent with previous small cohort studies suggesting that glycoprotein IIb/IIIa inhibitors, including tirofiban, may be feasible in selected patients undergoing endovascular treatment of ruptured aneurysms (Chalouhi et al., 2012), (Samaniego et al., 2019). The absence of a clear signal of excess clinically relevant bleeding may partly be explained by the pharmacological profile of tirofiban, including its short half-life and reversible platelet inhibition, which may allow more controllable antiplatelet management compared with oral antiplatelet agents (Simonato et al., 2022). Nevertheless, our results should not be interpreted as definitive evidence of safety or equivalence. The study was underpowered to detect small or moderate differences in rare but clinically important hemorrhagic complications, and the subgroup of patients receiving prolonged tirofiban was limited.

It is also important to consider tirofiban within the broader landscape of antiplatelet strategies in neurointerventional procedures. Other glycoprotein IIb/IIIa inhibitors, such as abciximab and eptifibatide, as well as the intravenous P2Y12 inhibitor cangrelor, are used in selected clinical settings (Simonato et al., 2022), (Hasanpour et al., 2024). The choice between these agents depends on local protocols, operator experience, aneurysm and device characteristics, bleeding risk, and the anticipated need for rapid reversibility. While our study focused specifically on tirofiban, the findings contribute to the broader question of how intravenous antiplatelet therapy can be used safely in complex ruptured aneurysm treatment.

An important aspect of the present study is the evaluation of prolonged intravenous tirofiban administration. In many healthcare systems, prolonged intravenous antiplatelet therapy is uncommon, and patients are typically transitioned to oral dual antiplatelet therapy shortly after intervention for practical, economic, and patient comfort reasons. In our cohort, prolonged tirofiban administration was used in selected complex cases, potentially reflecting delayed transition to oral therapy, ongoing neurocritical care requirements, or concern for delayed thromboembolic complications. Therefore, the longer hospitalization observed in this subgroup should be interpreted cautiously and may reflect disease severity and treatment complexity rather than an independent effect of tirofiban itself.

Beyond peri-interventional thromboembolic prevention, tirofiban has also been investigated as a potential strategy to reduce delayed cerebral ischemia after subarachnoid hemorrhage, possibly by targeting microthrombotic mechanisms involved in vasospasm and secondary ischemic injury (Zanaty et al., 2021), (Zanaty et al., 2020). This potential dual role — prevention of immediate thromboembolic complications and possible reduction of delayed ischemic events — is clinically interesting and warrants further investigation. However, our study was not designed to assess delayed cerebral ischemia, vasospasm, or functional neurological outcome, and no conclusions regarding these potential benefits can be drawn from the present data.

Several limitations must be acknowledged. First, the study was retrospective and monocentric, with a limited sample size. Although propensity score matching was used to reduce baseline differences, residual imbalance remained, and unmeasured confounding cannot be excluded. Second, tirofiban was administered for heterogeneous indications, including stent-assisted coiling, flow-diverter implantation, intrasaccular devices, and periprocedural thromboembolic complications. These scenarios differ substantially in procedural complexity, thromboembolic risk, bleeding risk, and expected need for antiplatelet therapy. Third, important variables such as aneurysm morphology, exact device type, timing and number of secondary neurosurgical procedures, vasospasm, delayed cerebral ischemia, and detailed antiplatelet transition strategies were not uniformly available across the study period.

The long inclusion period from 2006 to 2024 introduces additional potential treatment-era bias, as endovascular devices, antiplatelet protocols, imaging follow-up, and neurocritical care evolved substantially over time. Furthermore, outcome ascertainment was limited by missing or non-evaluable data for some endpoints. The analysis focused mainly on radiographic bleeding complications, hospitalization, and 30-day mortality, whereas functional outcomes were not systematically available. These limitations restrict the clinical interpretation of the findings.

In one patient, tirofiban had to be discontinued and replaced by dual antiplatelet therapy with aspirin and clopidogrel after the development of acute thrombocytopenia, a rare but known complication of tirofiban therapy (Jiménez-Rodríguez et al., 2023). Although such events were uncommon in our cohort, they underline the need for careful laboratory monitoring during treatment.

## 5 Conclusion

In conclusion, tirofiban administration after endovascular treatment of ruptured intracranial aneurysms was not associated with a statistically significant increase in spontaneous, surgery-associated, or clinically relevant secondary intracranial bleeding in this selected retrospective cohort. However, due to the retrospective design, limited sample size, heterogeneous treatment indications, residual confounding, and incomplete outcome data, these findings should be interpreted cautiously. Larger prospective multicenter studies with standardized antiplatelet protocols, imaging follow-up, platelet monitoring, and functional outcome assessment are needed to better define the bleeding risk, clinical benefit, and optimal duration of tirofiban therapy in this setting.

## Author contributions

Conceptualization, J.S. and M.G.; methodology, M.G., J.B., J.K., D.M.H., and C.M.; validation, J.S., T.B.B., and M.R.; formal analysis, M.G. and J.S.; resources, C.Z., M.W., T.B.B., and B.M.; data curation, M.G. and P.P.; writing—original draft preparation, M.G. and J.S.; writing—review and editing, T.B.B., M.W., and B.M.; visualization, M.G.; supervision, J.S.; project administration, J.S.

## Availability of data and materials

All source data are stored at the Department of Diagnostic and Interventional Neuroradiology, Technical University, Munich. We invite parties interested in collaboration and data exchange to contact the corresponding author directly.

## Funding

The author(s) received no financial support for the research, authorship, and/or publication of this article.

## Conflicts of interest

None.

## References

[bib1] Cagnazzo F., Di Carlo D.T., Petrella G., Perrini P. (2020). Ventriculostomy-related hemorrhage in patients on antiplatelet therapy for endovascular treatment of acutely ruptured intracranial aneurysms. A meta-analysis. Neurosurg. Rev..

[bib2] Chalouhi N., Jabbour P., Kung D., Hasan D. (2012). Safety and efficacy of tirofiban in stent-assisted coil embolization of intracranial aneurysms. Neurosurgery.

[bib3] Hasanpour M., Maleki S., Rezaee H., Aminzadeh B., Abbasi Shaye Z., Keykhosravi E. (2024). Glycoprotein IIb/IIIa inhibitors in the treatment of thromboembolic events related to endovascular treatment of cerebral aneurysms-systematic review and meta-analysis. NeuroRadiol. J..

[bib4] Hoh B.L. (2023). Guideline for the management of patients with aneurysmal subarachnoid hemorrhage: a guideline from the American heart Association/American stroke association,”. Stroke.

[bib5] Hudson J.S. (2018). Hemorrhage associated with ventriculoperitoneal shunt placement in aneurysmal subarachnoid hemorrhage patients on a regimen of dual antiplatelet therapy: a retrospective analysis. J. Neurosurg..

[bib6] Hudson J.S. (2019). External ventricular drain and hemorrhage in aneurysmal subarachnoid hemorrhage patients on dual antiplatelet therapy: a retrospective cohort Study. Neurosurgery.

[bib7] Jiménez-Rodríguez G.-M. (2023). Severe acute thrombocytopenia after treatment with tirofiban: a case series approach. Interv. Cardiol. Lond. Engl..

[bib8] Kim S., Choi J.-H., Kang M., Cha J.-K., Huh J.-T. (2016). Safety and efficacy of intravenous tirofiban as antiplatelet premedication for stent-assisted coiling in acutely ruptured intracranial aneurysms. AJNR Am. J. Neuroradiol..

[bib9] Limaye K. (2019). The safety and efficacy of continuous tirofiban as a monoantiplatelet therapy in the management of ruptured aneurysms treated using stent-assisted coiling or flow diversion and requiring ventricular drainage. Neurosurgery.

[bib10] Samaniego E.A. (2019). Safety of tirofiban and dual antiplatelet therapy in treating intracranial aneurysms. Stroke Vasc. Neurol..

[bib11] Schatlo B. (2021). Incidence and outcome of aneurysmal subarachnoid hemorrhage: the Swiss study on subarachnoid hemorrhage (swiss SOS). Stroke.

[bib12] Scholz C., Hubbe U., Deininger M., Deininger M.H. (2013). Hemorrhage rates of external ventricular drain (EVD), intracranial pressure gauge (ICP) or combined EVD and ICP gauge placement within 48 h of endovascular coil embolization of cerebral aneurysms. Clin. Neurol. Neurosurg..

[bib13] Simonato D. (2022). Glycoprotein IIb/IIIa inhibitors for the neurointerventionalist. Intervent Neuroradiol..

[bib14] von Elm E., Altman D.G., Egger M., Pocock S.J., Gøtzsche P.C., Vandenbroucke J.P. (2007). Strengthening the reporting of observational studies in epidemiology (STROBE) statement: guidelines for reporting observational studies. Br. Med. J..

[bib15] von Kummer R. (2015). The heidelberg bleeding classification: classification of bleeding events after ischemic stroke and reperfusion therapy. Stroke.

[bib16] Zanaty M. (2020). Tirofiban protocol protects against delayed cerebral ischemia: a case-series study. Neurosurgery.

[bib17] Zanaty M. (2021). Phase 1/2a trial of ISPASM. Stroke.

